# The Panaritium (Felon)—Consequences and Treatment

**Published:** 1872-09

**Authors:** Carl Proegler

**Affiliations:** Addison, Ill.


ARTICLE V.—*The Panaritium* (*Felon*)—*Consequences and Treatment.* By CARL PROEGLER, M.D., of Addison, Ill.

It may seem strange to write an elaborate article on the above subject, but as a learned and celebrated surgeon (Langenbeck) said: "Major surgery has often to show very little results, but minor surgery which has to deal with the every-day occurrences and accidents of life, is great in its results by the enormous number of small results it gains;" therefore I may be excused in writing about minor surgery to-day.

The real panaritium (in a narrower sense of the word), is an *acute inflammation which leads to suppuration, with an inclination to necrosis of the subcutaneous connective tissue, localized on the narrow space of the soft parts of the volar surface of the fingers and hand, but is also able to produce, by neglect and mismanagement, great suppuration, which by its results may destroy the function of the finger and even hand.* (Huetter.)

According to this definition the character of the panaritium lies in the acuteness and quick progress of the inflammation to suppuration.

The causes are numerous; and according to statistics and numerous observations the working classes of our population, and among these again those who use mostly their fingers, as maid-servants, joiners, carpenters, mechanics, etc., etc., contribute largely to our cliniques and surgeons. Therefore we are disposed to believe that the *panaritium is a traumatic inflammation; its peculiarities are mostly governed by the anatomical circumstances of the part on which the inflammation develops itself.*

I need not explain to physicians more at length the topographical anatomy of the hand, because I presume that every educated medical gentleman knows the relations of the bones, tendons, *fasciæ*, *vessels* and nerves. But I shall direct your attention to a peculiarity of construction of the soft parts on the volar surface of the finger.

The *subcutaneous connective tissue on the volar side of the finger and hand* differs from the subcutaneous connective tissue of the dorsal side and the remaining upper extremities, *by a strong development of thickness, by a construction of short and rigid connective tissue fibres*, which do not, as on nearly all other parts of the extremity, run parallel with the long axis of the extremity and connect the skin with the fasciæ in an acute angle, but, on the contrary, are drawn down perpendicularly by the papillar body.

By these anatomical relations, the skin is almost absolutely immovable on the deeper lying parts, while on the other side the skin on the dorsal surface of the hand and finger, fore and upper arm, can be moved without any difficulty for several lines on its base. In this peculiar construction of the subcutaneous connective tissue on the volar side, lies pretty much the whole secret of the panaritium.

Whether suppuration be the cause of an emigration of white-blood corpuscles or cellular proliferation, a swelling of the inflamed suppurative tissues will be always a companion of it. This swelling draws the blood and lymphatic vessels into connection. Their compression causes a collateral œdema, and when compression has risen to a certain point, the blood vessels become perfectly strangulated, and necrosis of tissues must necessarily ensue.

The rigidity of these parts is always very great, so that necrosis of the infiltrated connective tissue is most always present. Only a correct treatment may save these parts from local death ; sometimes the panaritium may end in an exfoliation of a necrosis, with pus-impregnated mass of connective tissue.

The presence of this necrosed tissue gives rise to more inflammation of the surrounding parts, and therefore nearly always extensive swellings are present, mostly on those places which are very much prone to swelling. On the finger these places are not the soft parts of the volar surface, but the soft parts of the dorsal surface, being farthest away from the original starting point of suppuration.

For young beginners cases of this kind will have something startling, if not well versed in their practice. You will behold a finger with a shiny, œdematous swelling of the skin and subcutaneous formation posteriorly, the volar surface but little swollen, but in reality the local symptoms are here to be found, and on the volar surface you will find them.

The pain, the true companion of the panaritium, is of an excruciating character, and Huetter, a German surgeon, says: "I do not wish anybody to make the experience on themselves of a panaritium, but if you ever have made it, then you will esteem the more the surgical therapeutics for such a complaint." As on the volar surface of the finger are an abundance of nerves, etc., the intensity of the pain may be easily conceived.

Fever is not always present, but very often a panaritium is ushered in by fever. Huetter explains this in the following manner: "The amount of pyrogone (fever-producing) substances is rather small, but resorption takes place in a relatively great measure, because the pyrogone substances of the pus stand from the moment of their formation under a very high pressure. This pressure caused by the rigid, short fibres of the volar subcutaneous connective tissue, forces the pyrogone substances into the root of the lymphatic vessels; reaching by this way the blood, its fever-producing qualities are revealed."

A teaspoonful of pus under the skin on the dorsal surface of the finger very rarely produces fever, but a few drops of it in the subcutaneous connective tissue of the volar surface are sufficient to raise the thermometer to 95 and even 100 degrees Fahrenheit.

And here I may remark that sometimes, *not often*, inflammation comes to a standstill, the advance of pus which surrounds the already mentioned necrosed mass, causes, at the height of the inflammatory stage, a perforation of the papillar bodies. A few drops of pus find their way into the rete malpighi, elevate the cuticle, a small vesicle opens and the pus escapes, pain ceases, granulations set in, the opening becomes larger, and by pressure the necrosed masses are forced through.

This is the favorable course the panaritium sometimes spontaneously takes, and even in this stage an active interference at the right place and in the right manner may shorten such spontaneous attacks.

The public at large has such a dread of "cutting" that very seldom does a case of panaritium present itself to the surgeon in the initial stage. Very often are the ravages so great that even a periosteal phlegmonous inflammation takes place. It appears generally on the finger as a suppurative synovitis of the sheath of the long flexor tendons, both sheath enveloping the flexor profundis and flexor sublimis. As soon as suppuration reaches this long synovial cavity, suppuration invades the whole cavity, involving even sometimes the whole hand.

It is well known that the felons of the thumb and little finger are the most dangerous, because the flexor pollicis longus has a long sheath which branches out to the root of the hand and the ligamentum carpi volare transversum.

[To be continued.]

